# Quantitative Ultrasound SWE of Carotid Plaque in Symptomatic and Asymptomatic Patients: A Systematic Review and Meta-Analysis

**DOI:** 10.3390/diagnostics16071085

**Published:** 2026-04-03

**Authors:** Salahaden R. Sultan, Faisal Albin Hajji, Muyaser Fatani, Ahmad Albngali, Abrar Alfatni, Amal Alsalamah, Reem T. Alturki, Abdullah M. Abdullah, Reda Jamjoom, Mohammad Khalil, Mohammed Alkharaiji, Adel Alzahrani

**Affiliations:** 1 Department of Radiologic Sciences, Faculty of Applied Medical Sciences, King Abdulaziz University, Jeddah 21589, Saudi Arabia; 2 Department of Radiology, King Abdulaziz University Hospital, King Abdulaziz University, Jeddah 21589, Saudi Arabia; 3 Department of Radiological Sciences, Batterjee Medical College, Jeddah 21442, Saudi Arabia; 4 Department of Radiological Sciences, College of Applied Medical Sciences, King Saud University, Riyadh 11362, Saudi Arabia; 5 Department of Surgery, Faculty of Medicine, King Abdulaziz University, Jeddah 21589, Saudi Arabia; 6 Department of Radiology, Faculty of Medicine, King Abdulaziz University, Jeddah 21589, Saudi Arabia; 7 Department of Public Health, College of Health Sciences, Saudi Electronic University, Riyadh 11673, Saudi Arabia; 8 Department of Diagnostic Radiology and Imaging, King Abdullah Medical City, Makkah 21955, Saudi Arabia

**Keywords:** ultrasound, SWE, elastography, carotid plaque, stroke

## Abstract

**Background/Objectives:** Ultrasound shear-wave elastography (SWE) enables quantitative assessment of tissue stiffness and may provide a non-invasive biomarker of carotid plaque vulnerability. This study aimed to evaluate the ability of SWE to differentiate symptomatic from asymptomatic carotid plaques and to assess its reproducibility. **Methods:** A systematic search of PubMed, Web of Science, EMBASE, and the Cochrane Library was conducted in the last ten years until January 2026 for publications evaluating carotid plaques using ultrasound SWE. Inclusion criteria required original publications that used quantitative ultrasound SWE parameters for the evaluation of carotid plaque in symptomatic and asymptomatic patients and/or investigated the reproducibility of SWE parameters for carotid plaques; non-carotid studies, non-original articles, and studies not comparing symptomatic versus asymptomatic plaques or not reporting reproducibility were excluded. Fourteen studies comprising 1781 carotid plaques were included. Quantitative SWE measurements were meta-analyzed using random effects. Differences between symptomatic and asymptomatic plaques were assessed using standardized mean differences (SMDs). The reproducibility of SWE measurements was evaluated using pooled correlation coefficients. Publication bias was evaluated using funnel plots and Egger’s regression test. **Results:** Ten studies including 1246 plaques compared SWE stiffness between symptomatic (*n* = 472) and asymptomatic plaques (*n* = 774). The meta-analysis demonstrated significantly lower stiffness values in symptomatic plaques compared with asymptomatic plaques (SMD −1.10, *p* < 0.001). Reproducibility analysis of correlation coefficients extracted from seven studies demonstrated excellent agreement for SWE measurements (r = 0.92, *n* = 602). Heterogeneity was observed across the included studies. No statistically significant evidence of publication bias was detected. **Conclusions:** Ultrasound SWE is a promising approach for assessing carotid plaque vulnerability, with lower SWE stiffness observed in symptomatic plaques compared to asymptomatic plaques. This finding should be interpreted with consideration of methodological heterogeneity and the cross-sectional nature of the available assessed evidence. Further prospective studies with standardized imaging protocols and longitudinal follow-up are needed to determine clinically applicable stiffness thresholds and evaluate the prognostic value of ultrasound SWE in cerebrovascular risk stratification.

## 1. Introduction

Ultrasound is a reliable imaging modality for vascular diagnostics, favoured for its non-invasiveness, affordability, and accessibility. B-mode ultrasound allows for morphological evaluation of carotid plaques, including their size, surface irregularity, and echogenicity. Grayscale median (GSM) analysis offers a quantitative measure of echogenicity, with low GSM values suggesting lipid-rich, echolucent plaques that are more prone to rupture [1,2,3]. Doppler ultrasound provides real-time hemodynamic data to estimate the degree of flow-limiting stenosis [4]. Contrast-enhanced ultrasound (CEUS) is promising for the visualization of intraplaque neovascularization, helping in differentiating between symptomatic and asymptomatic carotid plaques [5,6,7]. Elastography has emerged as an advance technology in ultrasound imaging by enabling non-invasive assessment of tissue stiffness [8,9]. Shear-wave elastography (SWE) offers a quantitative approach by generating shear waves via acoustic radiation force, tracking their propagation with ultrafast imaging, and calculating tissue stiffness from shear-wave velocity as Young’s modulus [10,11].

Ultrasound SWE has been applied successfully in a variety of clinical settings [8,12], and has been increasingly used as a promising non-invasive tool for characterizing carotid plaque composition and stability [13]. The rationale behind using elastography in carotid plaque is that higher stiffness regions often indicate stability, whereas lower stiffness values suggest vulnerable features [14,15]. Furthermore, YM values derived from SWE have demonstrated good correlation with GSM scores, reinforcing the technique’s potential to reflect both structural and compositional aspects of plaque pathology [16,17,18]. This study aims were to systematically review and meta-analyze the current evidence on the diagnostic performance of ultrasound SWE to differentiate between carotid plaques in symptomatic and asymptomatic patients, and to assess its reproducibility.

## 2. Methods

This study was conducted according to the Preferred Reporting Items for Systematic Reviews and Meta-Analyses (PRISMA) guidelines (Tables S1 and S2) [19].

### 2.1. Search Strategy and Study Selection

PubMed, Web of Science, EMBASE and Cochrane Library databases were searched for studies that potentially evaluated carotid plaques using SWE in the last ten years until January 2026. Search keywords used for searching titles and abstracts included the following: carotid artery OR carotid disease OR carotid atherosclerosis OR carotid plaque OR carotid plaque vulnerability OR carotid plaque neovascularization OR unstable carotid plaque OR symptomatic carotid artery plaque OR asymptomatic carotid artery plaque AND shear-wave elastography OR SWE OR ultrasound shear-wave elastography OR US-SWE OR two-dimensional shear-wave elastography OR 2D-SWE OR point shear-wave elastography OR pSWE. The search was restricted to ‘humans’ and ‘adults +19’ and ‘English’. Pre-specified inclusion criteria were used to prevent bias. The inclusion criteria were an original published paper that used quantitative ultrasound SWE parameters for the evaluation of carotid plaque in symptomatic and asymptomatic patients and/or investigated the reproducibility of SWE parameters for carotid plaques using correlation coefficients. Non-original articles including reviews, study protocols, pictorial essays, viewpoints and abstracts, non-human studies, and studies that did not provide SWE parameters of carotid plaques in symptomatic and asymptomatic patients, and did not assess the reproducibility of SWE parameters in assessing carotid plaque through correlation coefficients were excluded.

### 2.2. Data Acquisition

From the included studies, the following were extracted: study reference, primary aim, patient demographics and clinical information, sample size, reference standard test, ultrasound machine manufacturer and transducer, region of interest investigated for analysis, and study findings. For the meta-analysis, data provided on quantitative of SWE parameters were extracted and used to compare carotid plaques in symptomatic and asymptomatic patients, and to assess the reproducibility of elastography for the assessment of carotid plaque. Data were independently extracted from each eligible report by two groups of two reviewers each, with any discrepancies resolved through discussion and consensus with a third group of two additional reviewers. Symptomatic patients with carotid plaque were characterized as having the presence of neurologic symptoms in the ipsilateral carotid artery, and asymptomatic patients, on the other hand, were characterized with the absence of cerebrovascular events. In studies that included both symptomatic and asymptomatic patients and further classified plaques by echogenicity, echogenic plaques were considered stable, while echolucent plaques were classified as unstable [20]. SWE stiffness parameters were measured within the plaque ROI, including Young’s modulus in kPa and shear-wave velocity in m/s. The number of carotid plaques was considered the primary sample size; however, when this information was not reported in the published article, the number of patients included in the study was used instead. In studies reporting SWE measurements using different units (e.g., kPa and m/s), multiple quantitative parameters (e.g., average, maximum, or minimum), or both inter- and intra-observer correlations within the same study population, the total sample size was distributed approximately equally across the reported units and parameters to prevent duplication of participant data. If published studies further subdivided asymptomatic or symptomatic groups, or when SWE measurements were obtained from multiple regions of interest within the same plaque or from bilateral plaques in patients pre-classified as symptomatic or asymptomatic, the averaged values were calculated.

### 2.3. Quality

The quality and risk of bias of the included studies were evaluated using the Quality Assessment of Diagnostic Accuracy Studies tool [21] by three independent reviewers. Each study was assessed across several predefined criteria, with one point assigned for each criterion fulfilled. These criteria included: clearly defined patient selection criteria; inclusion of patients with carotid disease; availability of clinical information for the recruited participants; use a histopathology as a reference standard; independence of the reference standard from ultrasound findings; application of the same reference standard to all patients; verification of all samples using the reference standard; reporting the time interval between SWE assessment of carotid plaques and the reference test; description of the methods used for the reference standard; detailed SWE imaging protocol; specification of the SWE technique and ultrasound system used; blinding of outcome assessment; completeness of outcome data; and reporting and explanation of any subject withdrawals, when applicable.

### 2.4. Statistical Analysis

Data were grouped prior to analysis according to outcome measures (i.e., SWE values in symptomatic versus asymptomatic carotid plaques and the reproducibility of SWE in carotid plaque assessment). Each outcome was analyzed and presented using separate forest plots. SWE measurements of symptomatic and asymptomatic carotid plaques were analyzed and further subgrouped according to the reported measurement units (kPa or m/s). The mean and standard deviation of SWE values were used for quantitative synthesis. Studies reporting continuous outcomes as mean ± standard deviation (SD) were included directly; mean and SD were estimated is studies presenting data as median and interquartile range [22,23]. Continuous outcomes were pooled as standardized mean differences (SMDs) with 95% confidence intervals using a random-effects model, given the heterogeneity in elastography scales across the included studies. Statistical heterogeneity was assessed using the I^2^ statistic. Reproducibility outcomes were subgrouped into intra- and inter-observer agreement. Correlation coefficients (r) with corresponding 95% confidence intervals were used as the statistical estimates to assess the reproducibility of elastography in carotid plaque evaluation and were calculated. The strength of correlation was interpreted as follows: r < 0.50, poor; r = 0.50–0.75, moderate; r = 0.76–0.90, good; and r > 0.90, excellent [24]. Publication bias was evaluated using funnel plots and Egger’s regression test [25]. Stata/SE Statistical Software version 16.1 (StataCorp., College Station, TX, USA) was used for analysis and to create forest and funnel plots. Statistical significance was defined as *p* < 0.05.

## 3. Results

**Figure 1:**
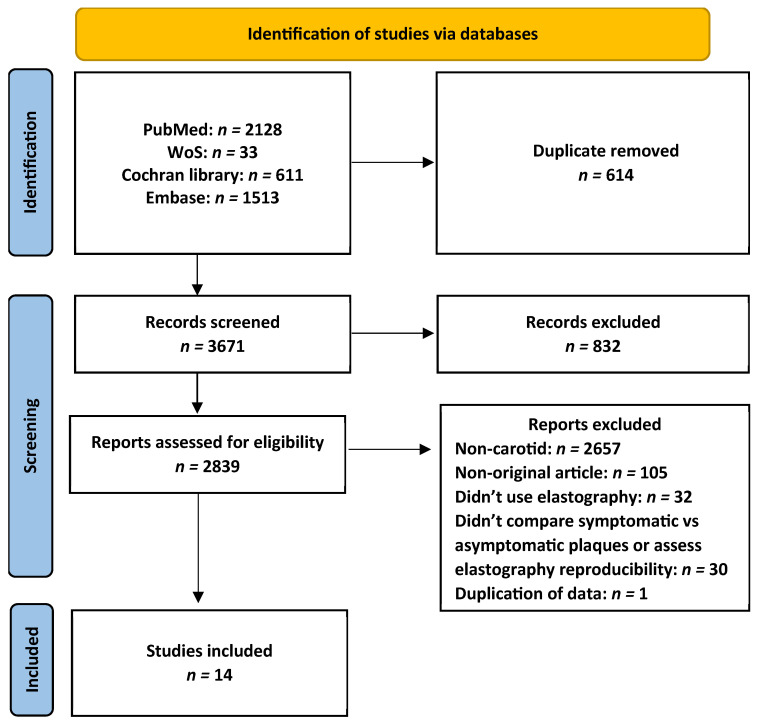
Flow chart for study retrieval and selection.

The database search identified 4285 publications from PubMed, Web of Science, EMBASE, and the Cochrane Library. After the removal of 614 duplicates, 3671 studies were screened by title and abstract. Of these, 832 publications were excluded as irrelevant. The remaining 2839 articles underwent full-text evaluation, and 2825 studies were excluded for the following reasons: non-carotid topic (*n* = 2657), non-original articles (*n* = 105), studies not using elastography (*n* = 32), studies not comparing symptomatic and asymptomatic plaques or not evaluating elastography reproducibility (*n* = 30), and duplicate data (*n* = 1). The study selection process is presented in Figure 1. The 14 included studies comprised 1781 carotid plaques. A summary of the included studies is shown in Table 1.

### 3.1. SWE in Symptomatic and Asymptomatic Carotid Plaques

**Figure 2:**
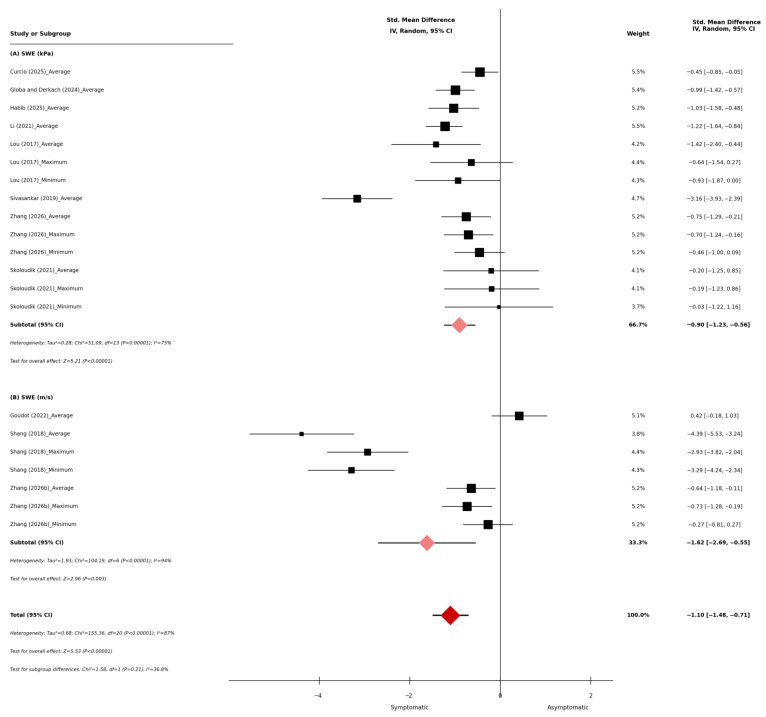
Shear-wave elastography (SWE) in symptomatic and asymptomatic carotid plaques.

Ten studies compared SWE stiffness measurements between symptomatic and asymptomatic carotid plaque groups, encompassing 1246 plaques (symptomatic, *n* = 472; asymptomatic, *n* = 774). Seven studies reported SWE values in kPa, two reported in m/s, and one provided measurements in both units (see Table 2 and Table 3). The average SWE values ranged from 12.4 to 81.1 kPa in symptomatic plaques and from 24 to 115.7 kPa in asymptomatic plaques. The maximum SWE values ranged from 31.2 to 159.6 kPa in symptomatic plaques and from 48.5 to 215.3 kPa in asymptomatic plaques, while the minimum SWE values ranged from 7.6 to 13.3 kPa in symptomatic plaques and from 9.5 to 26.7 kPa in asymptomatic plaques, while minimum values ranged from 7.6 to 13.3 kPa in symptomatic plaques and 9.5 to 13.8 kPa in asymptomatic plaques. In studies reporting SWE in m/s, the mean values ranged from 2.09 to 3.7 m/s in symptomatic plaques and 2.8 to 4.29 m/s in asymptomatic plaques. The maximum values ranged from 2.67 to 3.2 m/s in symptomatic plaques and 4.1 to 4.67 m/s in asymptomatic plaques, whereas the minimum values ranged from 1.51 to 1.7 m/s in symptomatic plaques and from 1.8 to 3.91 m/s in asymptomatic plaques. The overall meta-analysis demonstrated that SWE was significantly lower in symptomatic carotid plaques compared with asymptomatic plaques (standardized mean difference [SMD]: −1.10, 95% confidence interval [CI]: −1.48 to −0.71; *p* < 0.001, Figure 2). Subgroup analysis stratified by SWE measurement unit showed that studies reporting stiffness in kPa showed a significant reduction in symptomatic plaques (SMD: −0.90, 95% CI: −1.23 to −0.56; *p* < 0.001, Figure 2), and studies reporting shear-wave velocity in m/s similarly demonstrated lower values in the symptomatic group (SMD: −1.62, 95% CI: −2.69 to −0.55; *p* < 0.001, Figure 2). Statistically significant heterogeneity was observed across the overall analysis (I^2^ = 87%, *p* < 0.001, Figure 2).

### 3.2. Reproducibility of SWE in the Evaluation of Carotid Plaque Stiffness

**Figure 3:**
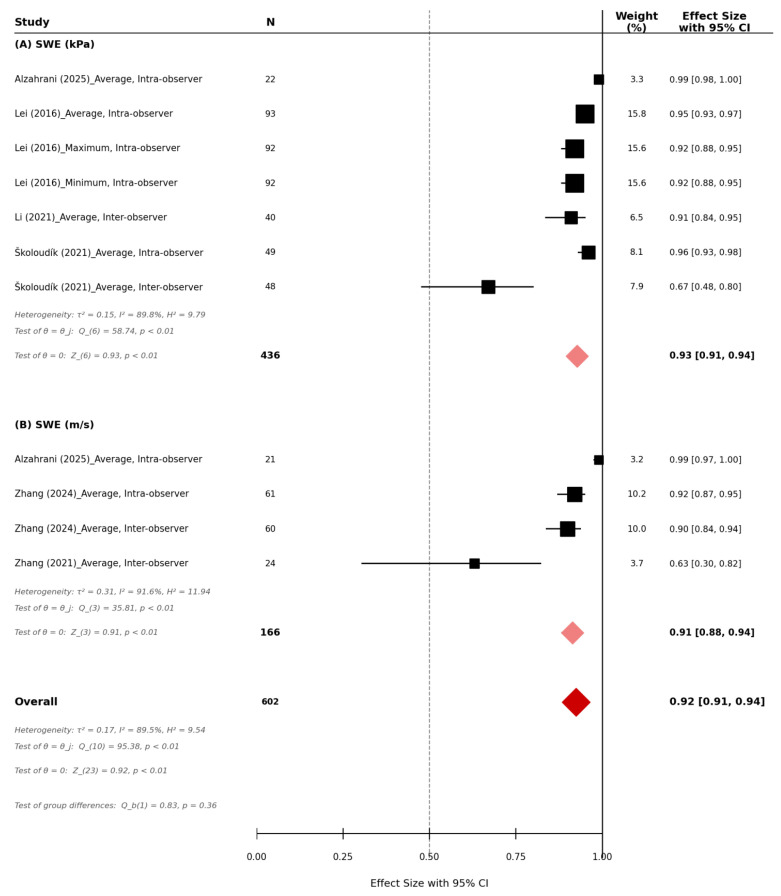
Reproducibility of shear-wave elastography (SWE) in carotid plaque stiffness assessment.

Five studies evaluated the reproducibility of SWE for carotid plaque stiffness assessment, comprising a total of 602 participants. The overall pooled reproducibility across all SWE measurements was excellent (r = 0.92, 95% CI 0.91–0.94, *p* < 0.01, *n* = 602, Figure 3). For studies reporting SWE stiffness in kilopascals (kPa), the pooled reproducibility demonstrated excellent agreement (r = 0.93, 95% CI 0.91–0.94, *p* < 0.01; *n* = 436, Figure 3). Individual studies demonstrated high reproducibility, with correlation coefficients ranging from 0.67 to 0.99. For studies reporting SWE as shear-wave velocity (m/s), pooled reproducibility also demonstrated excellent agreement (r = 0.91, 95% CI 0.88–0.94, *p* < 0.01; *n* = 166, Figure 3). Reported reproducibility correlations ranged from 0.63 to 0.99 across the included studies. Significant heterogeneity was observed across studies (I^2^ = 89.5%, *p* < 0.01, Figure 3).

### 3.3. Quality and Publication Bias

**Figure 4:**
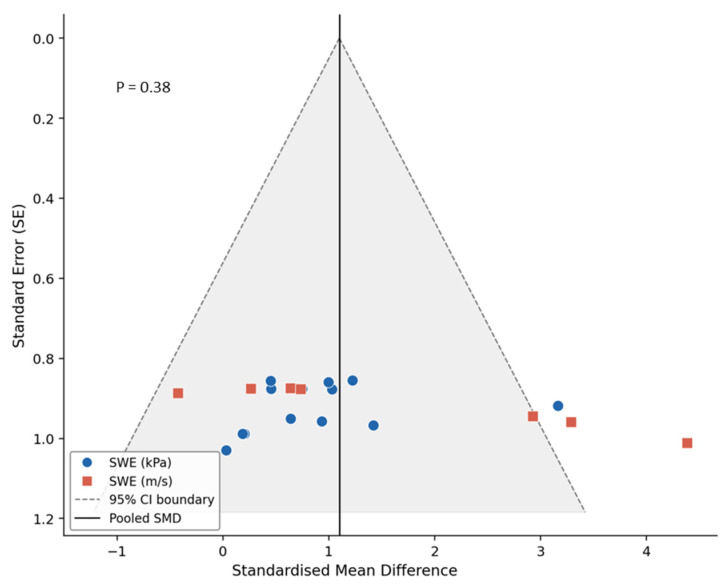
Funnel plot assessing publication bias in studies evaluating shear-wave elastography measurements of symptomatic and asymptomatic carotid plaque stiffness.

**Figure 5:**
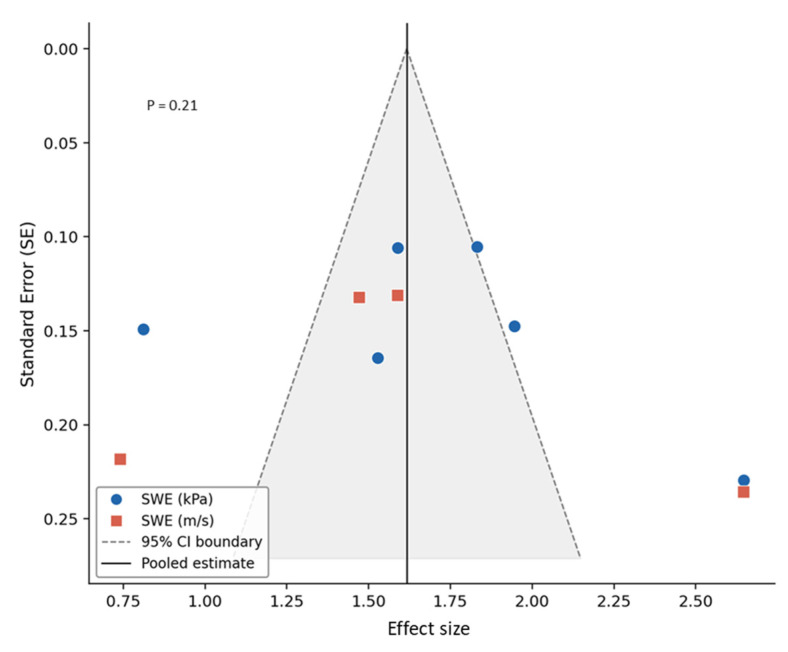
Funnel plot for the assessment of publication bias in studies evaluating the reproducibility of shear-wave elastography in carotid plaque stiffness measurement.

All studies recruited patients with carotid disease, clearly described patient selection criteria, provided sufficient clinical information, described the SWE imaging protocol, confirmed the specification of the SWE technique and ultrasound system used, and provided complete outcome data [13,16,17,18,26,27,28,29,30,31,32,33,34,35]. Eleven studies explained subject withdrawals [13,17,18,27,28,30,31,32,33,34,35], and eight reported blinding of assessment [13,16,17,18,28,31,32,33,34]. Three studies used histopathology as a reference standard independent of the SWE examination, applied the same reference standard to all patients, and verified carotid plaque vulnerability against that reference [27,30,33]. Two studies reported the period between the SWE evaluation and the reference test and described the methods for performing the reference standard [30,33]. For the comparison of SWE stiffness between symptomatic and asymptomatic carotid plaques, the funnel plot demonstrated a generally symmetrical distribution of studies around the pooled effect size, indicating no apparent small-study effects. This observation was supported by Egger’s regression test, which showed no statistically significant evidence of publication bias (*p* = 0.38, Figure 4). Similarly, for studies assessing the reproducibility of SWE measurements, visual inspection of the funnel plot revealed no clear asymmetry. Egger’s regression test likewise indicated no significant publication bias (*p* = 0.21, Figure 5).

## 4. Discussion

There has been increasing interest in the application of ultrasound SWE for the characterization of carotid plaque biomechanics and vulnerability. In this systematic review and meta-analysis, we evaluated the ability of SWE to differentiate between symptomatic and asymptomatic carotid plaques and assessed the reproducibility of SWE measurements in carotid plaque stiffness assessment. Based on the ranges reported across the included studies, the estimated midpoints of the reported SWE values are as follows: the approximate average stiffness was about 47 kPa in symptomatic plaques versus 70 kPa in asymptomatic plaques, with estimated maximum values of approximately 95 kPa and 132 kPa, respectively. Similarly, the estimated minimum stiffness values were approximately 10 kPa in symptomatic plaques and 18 kPa in asymptomatic plaques. In studies reporting shear-wave velocity, the estimated average velocity was about 2.9 m/s in symptomatic plaques and 3.5 m/s in asymptomatic plaques, with corresponding minimum values of approximately 1.6 m/s and 2.9 m/s, and maximum values of approximately 3.0 m/s and 4.4 m/s, respectively. The analysis demonstrated that SWE stiffness values were significantly lower in symptomatic plaques compared with asymptomatic plaques, and demonstrated excellent reproducibility for the assessment of carotid plaque stiffness. These findings highlight the potential clinical relevance of SWE in vascular surgical decision-making. While current indications for carotid intervention are primarily based on stenosis severity and symptom status, growing evidence highlights the importance of plaque vulnerability in determining cerebrovascular risk. The lower stiffness observed in symptomatic plaques suggests that SWE can identify mechanically vulnerable lesions beyond what conventional imaging alone provides. This may be particularly valuable in asymptomatic patients with moderate-to-severe stenosis, where reduced stiffness could indicate a higher biomechanical risk profile and support closer surveillance or earlier consideration of intervention. Accordingly, incorporating SWE into routine carotid ultrasound assessment may enhance risk stratification and assist in refining patient selection for surgical management.

SWE values demonstrated considerable variability across the included studies. The reported average stiffness values ranged widely, from 12.4 to 81.1 kPa and 1.51 to 3.6 m/s in symptomatic plaques and from 24 to 115.7 kPa and 1.8 to 4.67 m/s in asymptomatic plaques, reflecting both biological variability and different system and imaging pre-sitting requirements for carotid plaque assessment. The relationship between ultrasound SWE measurements and the histopathological characteristics of carotid plaques has been investigated, indicating that plaque stiffness assessed by SWE reflects the underlying tissue composition [14,15]. Higher Young’s modulus values have been reported in stable plaques compared with vulnerable plaques in patients undergoing carotid endarterectomy, with histological examination confirming that vulnerable plaques are characterized by larger necrotic cores, increased lipid content, and greater macrophage infiltration [27]. An association between shear-wave velocity and collagen composition within carotid plaques has been reported [33], highlighting the important role of collagen in determining plaque mechanical stiffness. Increased collagen content within the fibrous cap is generally associated with greater plaque stability by enhancing tensile strength and maintaining cap integrity, whereas reduced collagen content, increased collagen degradation, or thinning of the fibrous cap results in structurally weaker plaques that are more susceptible to rupture and therefore considered vulnerable [33,36]. These findings indicate that SWE-derived stiffness, though Young’s modulus and shear-wave velocity measurements, reflects the integrated biomechanical properties of plaque composition, supporting the potential role of SWE as a non-invasive imaging biomarker for the assessment of carotid plaque vulnerability. However, Goudot et al. (2022) reported no significant difference in overall stiffness between histologically defined vulnerable and stable plaques [30]. This may be explained by the intrinsic histological heterogeneity of atherosclerotic plaques, which often contain a mixture of lipid-rich necrotic cores, fibrous tissue, calcification, and intraplaque hemorrhage within the same lesion. The presence of multiple tissue components within the same plaque can result in overlapping elasticity values, thus affecting the ability of SWE to clearly differentiate vulnerable from stable plaques.

In addition to plaque characteristics and compositional features that may influence SWE measurements, variation between ultrasound imaging systems represents an important source of heterogeneity in the assessment of carotid plaque stiffness using shear-wave elastography (SWE). The included studies employed multiple ultrasound systems, which differ in hardware design, transducer characteristics, and proprietary signal-processing algorithms, factors that have been reported to influence shear-wave generation, detection, and stiffness estimation [37,38,39]. Imaging acquisition settings represent a factor that may influence the reported stiffness values of carotid plaques measured using SWE, even when the same ultrasound system is used. For example, among three studies employing the Canon Aplio system, two studies used a similar measurement scale (0–120 kPa) and reported lower stiffness values in symptomatic plaques compared with asymptomatic plaques: Habib et al. (2025) reported mean values of 34.2 kPa in symptomatic plaques and 64.8 kPa in asymptomatic plaques [26], whereas Li et al. reported 51.5 kPa and 78.5 kPa [31], respectively. The variation between these reported values may reflect differences in plaque composition and structural characteristics, from which a potential threshold of approximately 55–60 kPa may be considered as a range for differentiating symptomatic from asymptomatic carotid plaques. Values below this range may indicate softer, potentially vulnerable plaques, whereas values above it may correspond to more structurally stable plaques. A third study by Globa and Derkach (2024) used a broader measurement scale (0–180 kPa) on the imaging system, and reported higher stiffness values (69.6 kPa in symptomatic plaques and 83.58 kPa in asymptomatic plaques) [29], which may influence the potential range of values derived for this system for differentiating symptomatic from asymptomatic carotid plaques. An extended elastography scale enables visualization of highly stiff components, such as calcified regions within carotid plaques; however, it may reduce precision within the lower stiffness range that is more relevant for lipid-rich or haemorrhagic plaque components. In contrast, a narrower scale may compress stiffness differentiation at the upper end of the range and potentially reduce sensitivity for detecting heavily calcified regions, but it can provide improved colour resolution within the biologically relevant range associated with softer plaque components. Based on the stiffness distributions reported in the literature, an SWE scale of approximately 0–150 to 0–200 kPa and 0–5 m/s appears sufficient to capture the majority of biologically relevant carotid plaque stiffness values while maintaining adequate sensitivity for both soft and calcified plaque components. Furthermore, ROI placement represents an additional methodological factor that may influence SWE measurements and varied across the included studies. Most studies assessed plaque vulnerability in symptomatic and symptomatic patients and placed a single ROI encompassing the entire plaque, whereas one study sampled multiple regions within the plaque (see Table 1). Although using a single ROI on the entire plaque is operationally convenient and reproducible [16,18,28], considering a single average stiffness value across the entire plaque may mask the substantial structural heterogeneity of atherosclerotic lesions. Carotid plaques typically contain multiple components with distinct mechanical properties, including lipid-rich necrotic cores, fibrous tissue, calcified nodules, and intraplaque hemorrhage. Averaging stiffness across these heterogeneous regions may therefore obscure focal areas of low stiffness that represent mechanically weak zones and are closely associated with plaque vulnerability. These findings suggest that multi-region ROI sampling, potentially guided by the elastography stiffness map and B-mode imaging, may provide a more comprehensive assessment of plaque biomechanical heterogeneity by separately evaluating both low-echogenic, mechanically soft regions, which are often associated with a higher risk of plaque vulnerability, and high-stiffness, echogenic areas within the plaque. Together, the variability in SWE values reported across studies highlights the influence of technical factors that may affect the apparent stiffness values obtained and consequently limit direct numerical comparisons between studies. This variability also makes it challenging to establish a standardized cut-off value for differentiating symptomatic from asymptomatic carotid plaques. Therefore, any proposed thresholds should be interpreted cautiously.

There are several limitations that should be considered. Heterogeneity was observed across studies, which reflects differences in ultrasound imaging systems, elastography data acquisition and processing protocols, and measurement scales. Differences in ROI placement may have affected the reported stiffness values. Most studies measured stiffness across the entire plaque using a single ROI, which may not fully represent the structural heterogeneity of atherosclerotic plaques; thus, averaging stiffness across these heterogeneous regions may reduce the sensitivity of SWE to detect localized areas of mechanical weakness associated with plaque vulnerability. In addition, variability in ultrasound equipment and operator-dependent practices may further contribute to measurement inconsistency; specifically, the lack of standardized ROI sizing may lead to the inadvertent inclusion of adjacent normal tissue, potentially affecting stiffness estimates. In this review, all plaques reported in the included studies were incorporated into the analysis; however, in some studies involving patients with multiple plaques, the thickest plaque was selected as the target for SWE assessment, which may not necessarily ensure that the evaluated plaque is the one responsible for symptom development. Only three studies included histopathological validation of plaque composition, with most investigations relying on clinical symptom status as a surrogate marker of plaque vulnerability. While this approach is commonly used in clinical research, it limits the ability to directly correlate SWE measurements with underlying tissue characteristics. Future investigations focusing on patients with a single plaque and incorporating the validation of plaque vulnerability against histopathological reference standards are warranted, enabling a more precise characterization of the relationship between SWE measurements and clinical outcomes. The included studies were cross-sectional in design, with SWE measurements obtained after the occurrence of cerebrovascular symptoms in symptomatic patients. Prospective studies with larger cohorts, standardized imaging protocols, and longitudinal clinical follow-up are therefore required to further evaluate the potential of SWE stiffness measurements as predictors of subsequent cerebrovascular events.

## 5. Conclusions

Ultrasound SWE is a promising non-invasive imaging technique for differentiating symptomatic from asymptomatic carotid plaques, with symptomatic lesions demonstrating lower stiffness values. These findings support the ability of SWE to reflect biomechanical features associated with plaque composition and vulnerability, supporting its reliability as a quantitative imaging biomarker. SWE enables objective quantification of plaque biomechanical properties and may enhance current risk stratification approaches. This may be particularly valuable in asymptomatic patients with non-severe stenosis, where lower stiffness values could reflect a higher-risk plaque phenotype and support clinical and surgical decision-making. With further methodological standardization, SWE may evolve into a valuable adjunct to conventional carotid ultrasound for improved plaque characterization. Future longitudinal prospective studies are warranted to evaluate SWE in larger populations, particularly in asymptomatic individuals, to determine whether plaque stiffness measurements can help identify plaques at risk of becoming symptomatic and contribute to improved cerebrovascular risk stratification.

## Figures and Tables

**Table 1:** Summary of included studies listed from latest to earliest.

Reference (Author/Year)	Primary Aim	Sample Size (Symptomatic/Asymptomatic)	Histology as a Reference	ROI	Machine/Transducer	Findings
Zhang et al., 2026 [13]	To evaluate the efficiency of multimodal ultrasonography in symptomatic and asymptomatic carotid plaques as potential biomarkers of ischemic stroke	476 (105/371)	No	Entire plaque	Mindray Resona 9/Linear array 15-3 MHz	SWE demonstrated lower stiffness values in symptomatic carotid plaques compared with asymptomatic plaques
Habib et al., 2025 [26]	To identify differences in atherosclerotic plaque elasticity and plaque echogenicity among symptomatic and asymptomatic groups.	58 (29/29)	No	Entire plaque	Canon Aplio/Linear array 15-4 MHz	SWE can help to identify unstable plaque
Curcio et al., 2025 [27]	To study the ability of non-invasive methods, including SWE, for assessing the biomechanical features of vulnerable and stable plaques in patients undergoing CEA	100 (43/57)	Yes	Entire plaque	Esaote MyLabX8/Linear array 15-4 MHz	SWE stiffness measurements were able to differentiate patients with vulnerable plaques from stable plaques
Alzahrani et al., 2025 [18]	To assess carotid plaque stiffness in patients undergoing CABG using SWE, and its reproducibility	43	No	Maximum plaque thickness	Siemens Redwood/Linear array 10-4 MHz	SWE is a reproducible tool for quantifying carotid plaque stiffness, with significant correlation to GSM and reduced stiffness in CABG compared to non-CABAG patients
Zhang et al., 2024 [28]	To evaluate IPN and plaque elasticity In patients with asymptomatic carotid stenosis, and its reproducibility	121	No	Entire plaque	Phillips EPIQ/Linear array 15-4 MHz	SWE is reproducible and demonstrated significant predictive value for the risk of stroke in patients with asymptomatic carotid plaque
Globa and Derkach, 2024 [29]	To identify characteristics of carotid plaque using SMI and SWE in symptomatic and asymptomatic patients	96 (51/45)	No	Entire plaque	Canon Aplio/Linear array 15-4 MHz	The low stiffness of the plaque based on to SWE data can characterize the potential instability of carotid plaques
Goudot et al., 2022 [30]	To evaluate plaque stiffness and WSS as potential biomarkers of vulnerability	46 (29/17)	Yes	Entire plaque	Aixplorer SuperSonic/Linear array 10-2 MHz	SWE of plaque stiffness showed no significant differences between vulnerable and stable plaques
Li et al., 2021 [31]	To assess vulnerable plaques using advanced ultrasound techniques	123 (65/58)	No	Entire plaque	Canon Aplio/Linear array 15-4 MHz	SWE can identify plaque characteristics that are associated with ischemic events and potentially vulnerable plaque
Školoudík et al., 2021 [32]	To identify differences in atherosclerotic plaque elasticity measured using SWE among individuals with symptomatic and asymptomatic stable carotid plaques	97 (11/86)	No	Entire plaque	Aixplorer SuperSonic/Linear array 15-4 MHz	SWE is useful for differentiating stable from unstable atherosclerotic plaques in carotid arteries.
Zhang et al., 2021 [33]	To assess the association between IPN and plaque elasticity and to correlate ultrasound findings with histopathological features	94	Yes	Multiple regions within the plaque	Siemens Acuson/Linear array 9-4 MHz	SWE can evaluate the vulnerability of the carotid plaque by assessing the elasticity of the plaque
Sivasankar et al., 2019 [34]	To assess carotid plaque stiffness using SWE and to explore the association between stiffness metrics and plaque vulnerability	60 (30/30)	No	Multiple regions within the plaque	Siemens Acuson/Linear array 9-4 MHz	SWE is useful tool that can be used for the early detection of vulnerable carotid plaques
Shang et al., 2018 [17]	To evaluate the feasibility of SWE in assessing the stiffness of carotid plaque	129 (78/51)	No	Entire plaque	Aixplorer SuperSonic/Linear array 15-4 MHz	SWE can be used to evaluate the stiffness of carotid plaques, and shows an association with symptomatic ischemic stroke
Lou et al., 2017 [35]	To evaluate the feasibility of SWE of carotid plaques in patients presenting with cerebrovascular incidents	61 (31/30)	No	Entire plaque	Aixplorer SuperSonic/Linear array 15-4 MHz	SWE can evaluate carotid plaque stably and can detect symptomatic carotid plaques
Lei et al., 2016 [16]	To assess the carotid plaque stiffness using SWE and to evaluate its reproducibility	277	No	Maximum plaque thickness	Aixplorer SuperSonic/Linear array 10-2 MHz	SWE is a reproducible and reliable imaging method for the assessment of carotid plaque, providing information for the quantitative assessment of carotid plaque stiffness.

Abbreviation: CABG: coronary artery bypass; CEA: carotidendarterectomy; GSM: grayscale median; IPN: intraplaque neovascularization; SMI: superb micro-vascular imaging; SWE: shear-wave elastography; WSS: wall shear stress.

**Table 2:** SWE stiffness mean values in kPa of carotid plaques in symptomatic and asymptomatic patients reported across the included studies.

Reference (Author/Year)	SWE Value in Symptomatic (kPa)		SWE Value in Asymptomatic (kPa)	
Zhang et al., 2026 [13]	Average	18.9	Average	24
	Maximum	31.2	Maximum	48.5
	Minimum	7.6	Minimum	9.5
Habib et al., 2025 [26]	Average	34.2	Average	64.8
Curcio et al., 2025 [27]	Average	12.4	Average	34.7
Globa and Derkach, 2024 [29]	Average	69.6	Average	83.58
Li et al., 2021 [31]	Average	51.5	Average	78.5
Školoudík et al., 2021 [32]	Average	36.4	Average	41.3
	Maximum	59.1	Maximum	66.5
	Minimum	13.3	Minimum	13.8
Sivasankar et al., 2019 [34]	Average	30.5	Average	42.2
Lou et al., 2017 [35]	Average	81.1	Average	115.7
	Maximum	159.6	Maximum	215.3
	Minimum	11.1	Minimum	26.7

Abbreviation: SWE: shear-wave elastography.

**Table 3:** SWE stiffness mean values in m/s of carotid plaques in symptomatic and asymptomatic patients reported across the included studies.

Reference (Author/Year)	SWE Value in Symptomatic (m/s)		SWE Value in Asymptomatic (m/s)	
Zhang et al., 2026 [13]	Average	2.5	Average	2.8
	Maximum	3.2	Maximum	4.1
	Minimum	1.7	Minimum	1.8
Goudot et al., 2022 [30]	Average	3.6	Average	3
Shang et al., 2018 [17]	Average	2.09	Average	4.29
	Maximum	2.67	Maximum	4.67
	Minimum	1.51	Minimum	3.91

Abbreviation: SWE: shear-wave elastography.

## Data Availability

The data presented in this study are included in the article. Further inquiries can be directed to the corresponding author.

## Supplementary Materials

The following supporting information can be downloaded at: https://www.mdpi.com/article/10.3390/diagnostics16071085/s1, Table S1: PRIMSA 2020 checklist. Table S2: PRISMA 2020 abstract checklist.
